# Supplementary material for: Insights into the limited global spread of the immune evasive SARS-CoV-2 variant Mu
Source: medRxiv. 2022 Mar 30:2022.03.28.22273077. Preprint. [Version 1] doi: 10.1101/2022.03.28.22273077 (PMC8978943; doi:10.1101/2022.03.28.22273077)
Supplement: 1 [file NIHPP2022.03.28.22273077V1-supplement-1.pdf]

medRxiv preprint doi: https://doi.org/10.1101/2022.03.28.22273077; this version posted March 30, 2022. The copyright holder for this preprint (which was not certified by peer review) is the author/funder, who has granted medRxiv a license to display the preprint in perpetuity.
It is made available under a CC-BY 4.0 International license .

SUPPLEMENT

Supplementary Figure 1: Intrinsic replication of SARS-CoV-2 VOCs in Calu-3 cells.

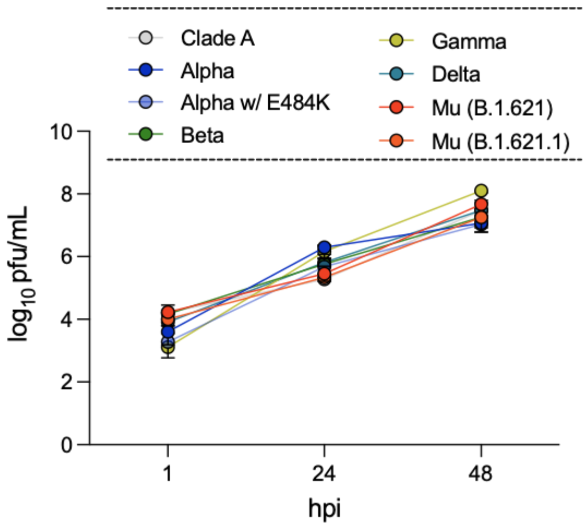

medRxiv preprint doi: https://doi.org/10.1101/2022.03.28.22273077; this version posted March 30, 2022. The copyright holder for this preprint (which was not certified by peer review) is the author/funder, who has granted medRxiv a license to display the preprint in perpetuity.
It is made available under a CC-BY 4.0 International license .

Supplementary Table 1: Lineage distribution for Figure 2a.

Lineage	Count
Mu	1957
B.1.621	1373
B.1.621.1	378
B.1.621.2	62
BB.2	144
VOCs (Alpha, Beta, Gamma, Delta, Omicron)	9
B.1.546	211
B.1.618	132
B.1.625	167
References	2
Total	*2478*

Supplementary Table 2: Number of Mu and total SARS-CoV-2 genome sequences submitted to GISAID by geographic region

Region/Continent	No. Mu genomes reported	No. total genomes reported	Submitting labs per 1M population
North America	6232	2511434	1.36
Central America	913	73699	0.45
South America	6676	162954	0.54
Europe	1197	3629925	1.95
Africa	1	70808	0.16
Asia	31	450875	0.12

Supplementary Table 3: Values for Fig. 2c

Region	Mean days to submission (Mu)	Mean days to submission (Delta)
North America	71.78709	30.72636
Central America	54.13363	34.0446
South America	42.30501	39.44826
Colombia	85.41713	57.31864

medRxiv preprint doi: https://doi.org/10.1101/2022.03.28.22273077; this version posted March 30, 2022. The copyright holder for this preprint (which was not certified by peer review) is the author/funder, who has granted medRxiv a license to display the preprint in perpetuity.
It is made available under a CC-BY 4.0 International license .

Europe	43.68212	16.52821
Asia	123.41935	57.74474

Supplementary Figure 2: Frameshift in ORF3 results in a premature stop codon in the Mu genome.

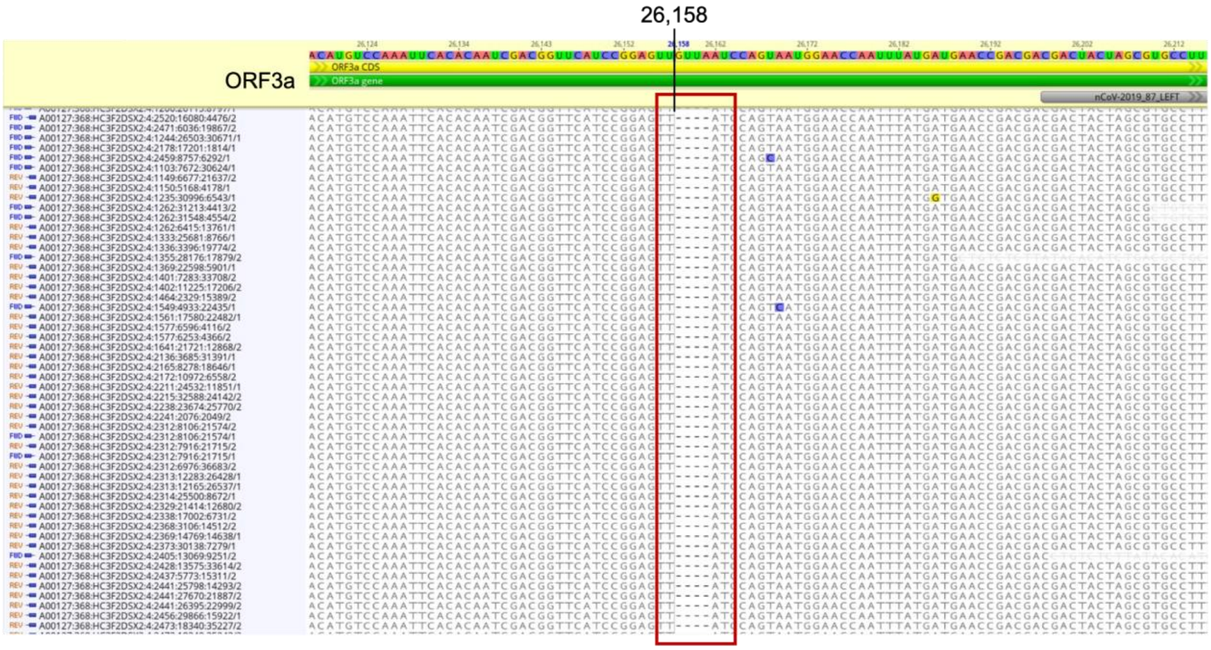

Supplementary Figure 3: Time-dependent variant frequencies by continent. Variant frequencies between October 2020 and December 2021 based on genome sequences available on GISAID.org as of January 19, 2022. Graphs rendered in Prism v.9.3.1.

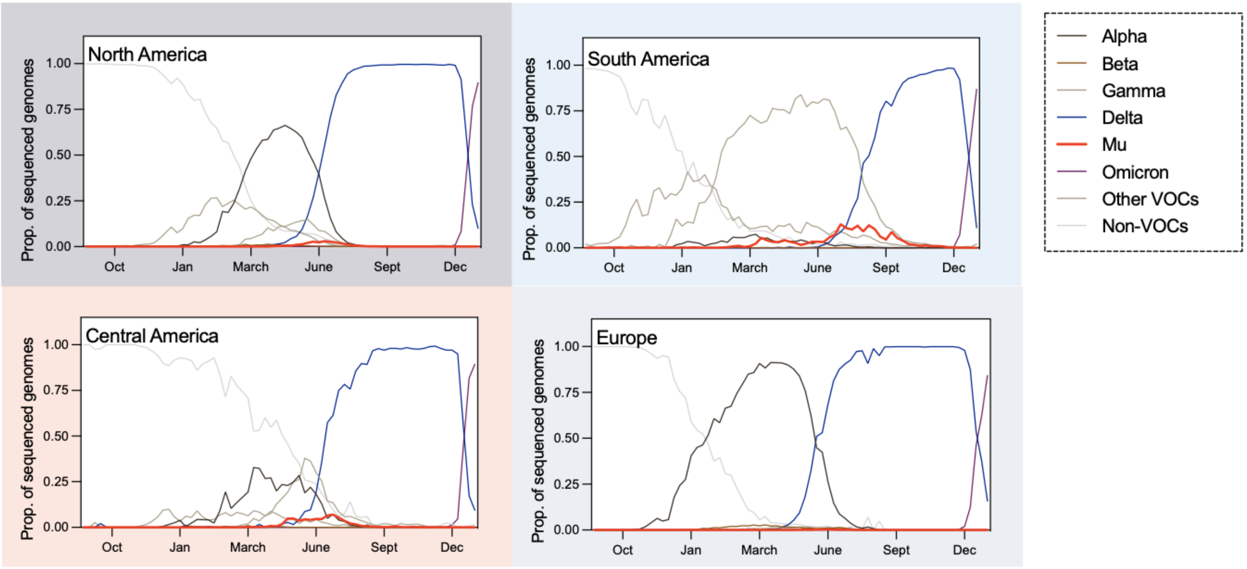

Supplementary Table 4: Lineages assigned to variant categories for frequency plots

Variant category	Lineages

medRxiv preprint doi: https://doi.org/10.1101/2022.03.28.22273077; this version posted March 30, 2022. The copyright holder for this preprint (which was not certified by peer review) is the author/funder, who has granted medRxiv a license to display the preprint in perpetuity.
It is made available under a CC-BY 4.0 International license .

Alpha	B.1.1.7, Q.X
Beta	B.1.351, B.1.351.X
Gamma	P.1, P.1.1, P.1.2
Delta	B.1.617.2, AY.X
Mu	B.1.621, B.1.621.X, BB.X
Omicron	B.1.1.529, BA.X
other-VBMs	B.1.427, B.1.429, B.1.525, B.1.526, B.1.617.1, B.1.617.3, P.2, C.37, C.37.1
non-VBMs	Remaining lineages not listed above
